# Pyroptosis: a double-edged sword in lung cancer and other respiratory diseases

**DOI:** 10.1186/s12964-023-01458-w

**Published:** 2024-01-15

**Authors:** Xiao Liang, Ya Qin, Dan Wu, Qiong Wang, Hongshuai Wu

**Affiliations:** 1 Department of Oncology, the Affiliated Jiangyin Hospital of Nantong University, 163# Shoushan Road, Jiangyin, Jiangsu 214400 P. R. China; 2 Wuxi Key Laboratory of Biomaterials for Clinical Application, Department of Central Laboratory, the Affiliated Jiangyin Hospital of Nantong University, 163# Shoushan Road, Jiangyin, Jiangsu 214400 P. R. China

**Keywords:** Pyroptosis, Caspase, Gasdermin, Lung cancer, Pneumonia

## Abstract

Pyroptosis is an active cell death process mediated by gasdermin family proteins including Gasdermin A (GSDMA), Gasdermin B (GSDMB), Gasdermin C (GSDMC), Gasdermin D (GSDMD), Gasdermin E (GSDME, DFNA5), and DFNB59. Emerging evidences have shown that pyroptosis contributes to many pulmonary diseases, especially lung cancer, and pneumonia. The exact roles of pyroptosis and gasdermin family proteins are tremendously intricate. Besides, there are evidences that pyroptosis contributes to these respiratory diseases. However, it often plays a dual role in these diseases which is a cause for concern and makes it difficult for clinical translation. This review will focus on the multifaceted roles of pyroptosis in respiratory diseases.

## Introduction

The scope of cell death has been greatly expanded in recent years. More molecularly oriented definitions of terms including necroptosis, mitochondrial permeability transition (MPT)-driven necrosis, ferroptosis, parthanatos, entotic cell death, NETotic cell death, lysosome-dependent cell death, autophagy-dependent cell death, immunogenic cell death, cellular senescence, and mitotic catastrophe [1–5]. Pyroptosis is an active programmed cell death process with a strong inflammatory response and manifests as cell membrane pore formation, which eventually leads to chromatin fragmentation, cell swelling, and plasma membrane lysis [6]. At a molecular level, the gasdermin protein family plays an important role in cell membrane pore formation and the activation of pyroptosis [7]. As a large family, gasdermin has six members: Gasdermin A (GSDMA), Gasdermin B (GSDMB), Gasdermin C (GSDMC), Gasdermin D (GSDMD), Gasdermin E (GSDME, DFNA5), and DFNB59 in the human genome [7]. Initially, pyroptosis was discovered to be involved in immune defense against infections [8]. However, the role of pyroptosis soon spread to many other diseases including some respiratory diseases. With further study, more modes of pyroptosis have been explored in pulmonary diseases [9]. In this review, we provide a detailed discussion of the double-edged role of pyroptosis in the regulation of pulmonary diseases and the challenges encountered in clinical translation.

### Pyroptosis

**Fig. 1:**
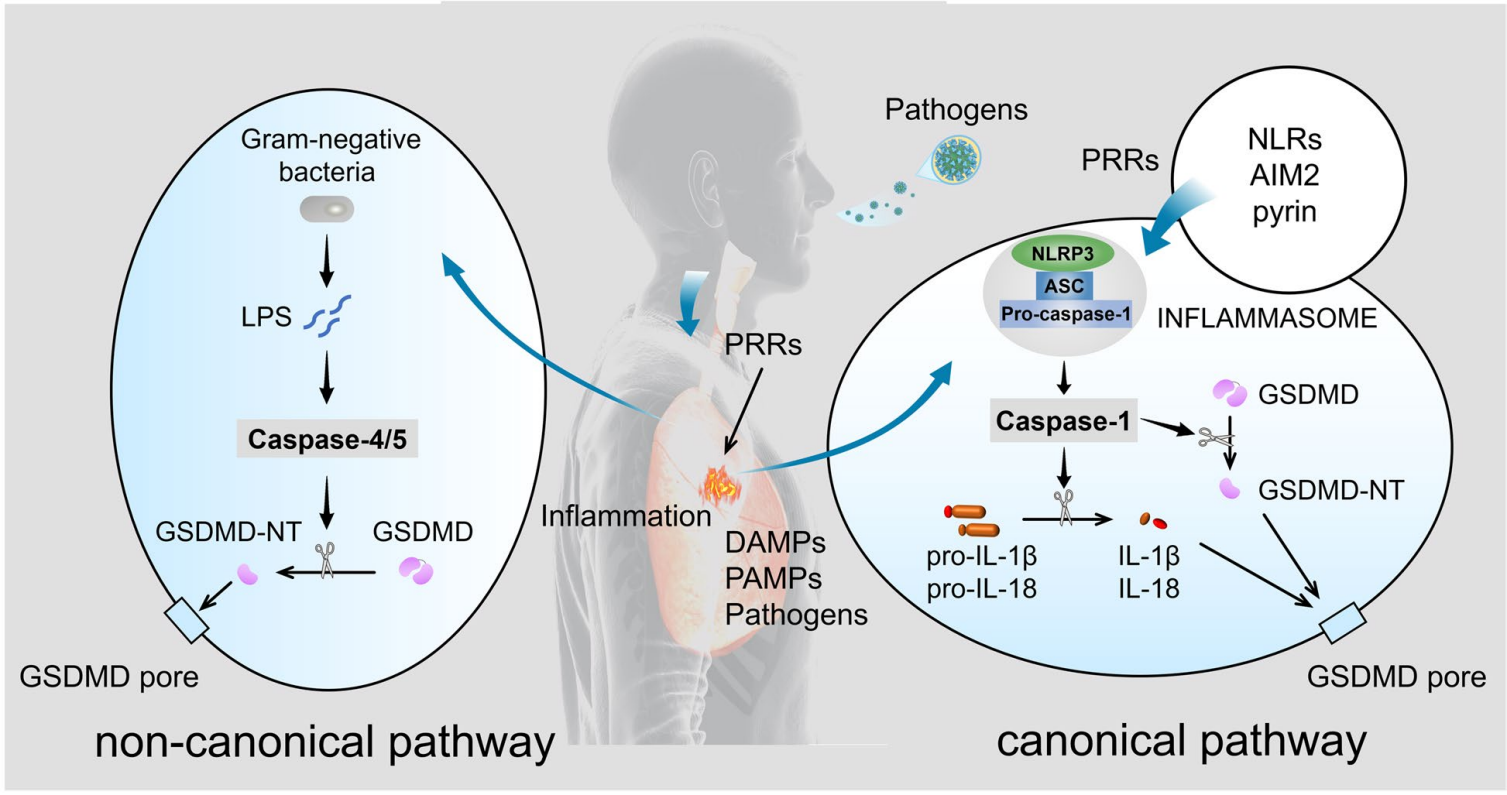
Activation mechanism of pyroptosis in the respiratory system in the canonical pathway and the non-canonical pathway. In canonical pathway, the NLRP3 inflammasome is taken as a representative example. The NLRP3 inflammasome consists of NLRP3, an important member of NLRs (NOD-like receptors), ASC, and pro-caspase-1. In addition to NLRs and pyrin, absent in melanoma 2 (AIM2) can also form inflammasomes to activate pyroptosis

In 1992, an atypical form of cell death was observed in macrophages [10]. A subsequent study revealed that caspase-1 activation was involved in this cell death [11]. In 2001, caspase-1-dependent cell death was identified as a proinflammatory programmed event and named pyroptosis by Brad T. Cookson and Molly A. Brennan [12]. Caspase-1 activation prompts the transition of pro-interleukin (IL)-1β to mature IL-1β and induces the production of IL-1β and IL-18 [13, 14]. IL-1β and IL-18 are finally released outside cells and induce strong inflammatory responses [8]. This caspase-1-mediated pyroptosis is referred to as canonical pyroptosis (Fig. 1). AS a multi-protein complex, the inflammasome plays a central role in the inflammatory response. It assembles in response to pathogen-associated molecular patterns (PAMPs) and danger-associated molecular patterns (DAMPs). The assembly and activation of inflammasomes are an important step in initiating canonical pyroptosis by inducing the self-cleavage and activation of caspase-1 [6]. The context of pyroptosis is expanded when caspase-11 was found to trigger a kind of mouse macrophage death that resembled the cell death induced by caspase-1 [15]. Caspase-4/5 was also found to have a similar function with the caspase-11 which is homologous to caspase-4/5. Unlike caspase-1 activated by ligands of inflammasomes, Caspase-4/5/11 activated by cytosolic lipopolysaccharide (LPS) induces pyroptosis in a noncanonical pathway. The noncanonical pathway is suggested to play an important role in cell immunological responses to intracellular Gram-negative bacteria and some metabolic diseases associated with mitochondrial dysfunction (Fig. 1) [6].

The identification of the gasdermin family yields insight into the mechanism of pyroptosis. The activation mechanism of GSDMD has been preliminarily unmasked, while those of others are largely unknown [7]. GSDMD plays an important role in either the canonical pathway or the non-canonical pathway. Danger signals or microbial infection can activate Caspase1/4/5/11. When activated, caspase1/4/5/11 can specifically cleave their direct substrate GSDMD into two parts: the C-terminal domain of GSDMD (GSDMD-CT) and the N-terminal domain of GSDMD (GSDMD-NT), a conserved domain [16]. GSDMD-NT triggers pyroptosis and GSDMD-CT inhibits the activation of GSDMD-NT by folding back on it. GSDMD-NT has the ability to connect to phosphoinositides and cardiolipin [16]. With oligomerization and membrane binding, GSDMD-NT leads to the lysis of membranes or the leakage of liposomes and forms pores in the membrane [16–18]. Subsequent alteration of osmotic pressure eventually causes cell swelling and lysis. The distribution of phosphoinositides is not symmetric on the plasma membrane. Therefore, GSDMD causes only lysis and leakage from within cells. IL-1β, as an important response of inflammasome activation, has also been demonstrated to be affected by GSDMD in some studies. GSDMD impacts the release of mature IL-1β, but not its maturation [16].

**Fig. 2:**
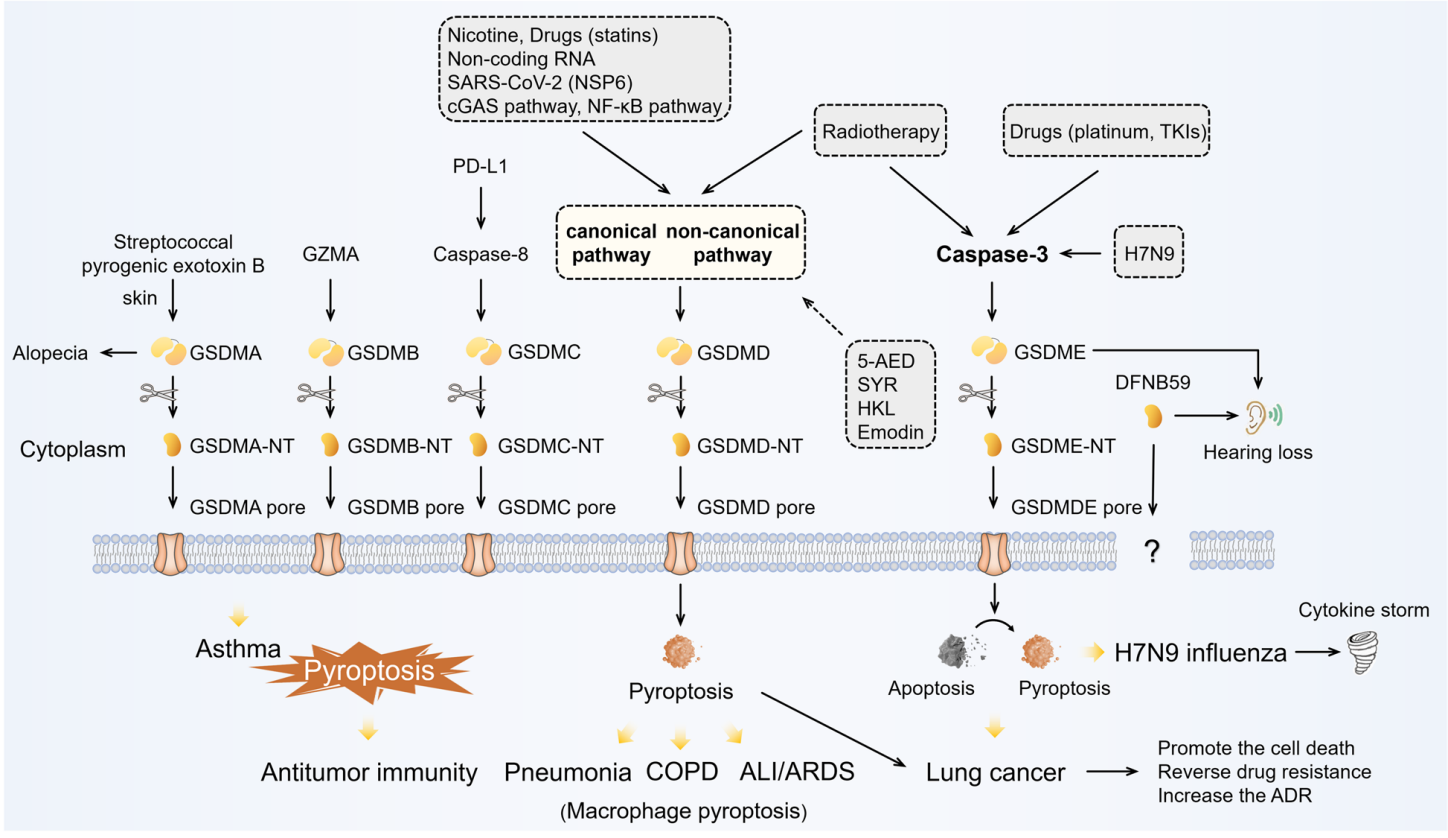
Pyroptotic pathway. GSDM superfamily members have six members: Gasdermin A (GSDMA), Gasdermin B (GSDMB), Gasdermin C (GSDMC), Gasdermin D (GSDMD), Gasdermin E (GSDME, DFNA5), and DFNB59. Gasdermins except for DFNB59 have similar architecture of two domains: an N-terminal and a C-terminal. N-terminal domains have pore-forming activities and activate pyroptosis. Pyroptosis has been reported to be involved in many pulmonary diseases. (Solid line: promoting effect, dotted line: inhibiting effect)

Other gasdermins have a similar architecture of two domains with GSDMD, except for DFNB59. The N-terminal domain of these GSDMD-like members can induce pyroptosis [7]. However, they lack caspase1/4/5/11 cleavage sites and their activation mechanisms are still unknown [16, 19]. Gasdermins function in the respiratory system, even with high epithelial expression specific to the skin and gastrointestinal tract [20]. Polymorphisms of GSDMB have been linked to some chronic inflammatory diseases in the respiratory tract, such as asthma [21]. GSDMC has also been implicated in lung cancer progression [22]. A new biological function of GSDMC was also revealed. Under hypoxia and TNFα treatment, GSDMC can be cleaved by caspase-8 mediated by nuclear programmed cell death 1 (PD1), which enhances GSDMC expression and switches apoptosis to pyroptosis [23]. GSDME was originally identified as DFNA5, a deafness gene and now seems to have many penetrated aspects of lung cancer. GSDME works as a mediator of p53 and has a potential role as a tumor suppression gene [24]. Methylation modification of GSDME is common in cancers that silence its expression [25]. Recently, some studies have reported that GSDME can be cleaved by caspase-3 specifically and switch caspase-3-mediated apoptosis to pyroptosis in many cases [26, 27]. Caspase-3 and GSDME work as a “switch” to shift cell death from apoptosis to pyroptosis (Fig. 2).

### Association between pyroptosis and pulmonary diseases

Respiratory diseases are one of the most important causes of global population death and mainly include inflammatory diseases, neoplastic diseases, and some autoimmune diseases [28–30]. Emerging evidence shows that pyroptosis contributes to these respiratory diseases [6]. However, it often plays a dual role in these diseases. Here, we provided some evidence and views of controversial pyroptosis in different pulmonary diseases.

### Lung cancer

The relationship between proinflammatory pyroptosis and lung cancer appears remarkably complicated [9]. Inflammation has been associated with the development and progression of various types of cancer. However, there are complex interactions between inflammatory processes and tumor development or progression [31, 32]. Recently, numerous studies have also found that promoting pyroptosis can inhibit tumor growth and reverse drug resistance [33]. In the adverse reactions related to tumor treatment, pyroptosis plays a role in promoting adverse reactions. Accumulated evidence indicates that pyroptosis exhibits a dual nature in lung cancer and its treatment.

Inflammation is the established factor in carcinogenesis of lung cancer [34]. Inflammasome proteins, including NLRP3 and AIM2, contribute to tumorigenesis by modulating immunity and cross-talk between the microenvironment and lung epithelial cells [35]. Additionally, some pyroptosis can occur in the tumor immune microenvironment through the caspase-1/GSDMD pathway, which will promote numerous malignant phenotypes in tumors including migration, invasion, and metastasis [36]. On the other hand, activated NLRP3 inflammasome aggregation of Ca^2+^ and the generation of reactive oxygen species (ROS) can promote pyroptosis and inhibit the proliferation of non-small cell lung cancer (NSCLC) [37]. Non-canonical pathway activated by caspase-4 has also been reported to eliminate NSCLC cells in vivo and in vitro [38].

Defects in apoptosis are known to be the overarching reason for the failure of anti-tumor treatment [39, 40]. With deeper research, we have found that pyroptosis is also involved in many anti-tumor treatments and plays a role in enhancing treatment efficacy [41]. Many chemotherapy drugs were reported to induce GSDME-mediated pyroptosis. After receiving chemotherapy, caspase-3 cleaves GSDME to generate a GSDME-N fragment and switches apoptosis to pyroptosis. Apoptotic appearance is overridden by pyroptosis in the case of high GSDME expression [27]. This phenomenon is observed in lung cancer treated with paclitaxel and cisplatin [41]. Pyroptosis induced by cisplatin seems to be higher than that induced by paclitaxel. It has also been reported that lower GSDME expression has an appreciable correlation with worse outcomes for NSCLC patients treated with platinum. GSDME-mediated pyroptosis may increase the sensitivity of platinum by promoting T cell infiltration [42]. The efficacy of platinum could also be enhanced by combination with BI2536, a PLK1 kinase inhibitor, by inducing pyroptosis and affecting DNA damage repair [43]. Some noncoding RNAs can also sensitize chemoresistant cancer cells to cisplatin by activating pyroptosis [44]. Additionally, GSDME executes caspase-3-meditated pyroptosis in EGFR-altered, ALK-rearranged, and KRAS-mutant tumors with genotype-matched regimens. The co-occurrence of pyroptosis and apoptosis contributes to the response to TKIs [45]. However, this pyroptosis appeared to marginally affect the treatment efficacy. In addition to the uncertain therapeutic effects of pyroptosis, it still has other concerning dual aspects. GSDME-mediated pyroptosis can act on normal tissues and exacerbate chemotherapy-induced toxicity. GSDME knockout can attenuate cisplatin-induced acute kidney injury and weight loss [27, 46]. Inhibiting pyroptosis can also reduce drug-induced nausea and vomiting caused by cisplatin and can decrease GSDME-mediated intestinal epithelial cell death through the regulation of ROS/JNK/Bax signaling pathway [47]. Additionally, in therapy-induced liver damage and myocardial injury, reducing pyroptosis was observed to alleviate these treatment-related toxicities [48].

Pyroptosis, as a highly inflammatory cell death, is also involved in radiation-induced tissue damage. Increasing caspase-1 activation is observed in marginal zone cells of radiotherapy [49]. GSDMD and inflammasomes such as AIM2 and NLRP3 are involved in this pyroptosis [49, 50]. Sepsis can also be promoted by caspase-11 mediated pyroptosis [51]. Cyclic GMP-AMP synthase (cGAS) influences caspase-11 in a non-canonical pathway of pyroptosis to aggravate this life-threatening complication in radiotherapy [52]. Other studies have also reported that GSDME expression enhances the sensitivity of cancer cells to radiation treatment by recruiting and activating NK cells to enhance antitumor immunity [53]. In addition, this GSDME-mediated pyroptosis induces radiation-related toxicity. Knocking GSDME protects against tissue damage and weight loss [53].

Pyroptosis makes important contributions to the transformation of cold tumors into hot tumors [54]. Many inflammatory cytokines and DAMPs are secreted during the processes of pyroptosis such as HMGB1, IL-1β, and IL-18. Inflammatory cytokines, including IL-1β and IL-18, are secreted through the GSDM-mediated pore. IL-1β can activate CD8 + T cells and promote the generation of Th1 CD4 + T-cells [55, 56]. IL-18 can also play immunoregulatory roles in inducing Interferon-γ (IFN-γ), polarizing Th1 cells and recruiting and activating natural killer (NK) cells [57]. Unlike cytokines secreted through traversing the GSDM-mediated pore, HMGB1 is regulated indirectly by GSDMD [58]. It could increase related molecules required for CD8 + T-cell priming and migrate tumor-infiltrating to draining lymph nodes to increase anti-tumor T cell responses [59]. These findings explain why pyroptosis elicits alterations in the tumor microenvironment which plays a crucial role in cancer progression and response to therapy. According to a recent study, a new mechanism of GSDMB in immunotherapy is revealed. Specifically, IFNγ can upregulate GSDMB expression and lymphocyte-derived granzyme A (GZMA) can cleave it. This result provides a new insight into enhancing antitumor immunity [60]. Notably, cytokines including IL-18 and IL-1β, which is recognized as effector molecules of pyroptosis, play a various function in different microenvironments. This introduces a lot of uncertainty in the clinical application of pyroptosis.

Increasing evidence suggests that inhibiting pyroptosis can, to some extent, control tumor progression. However, it is still premature to determine that pyroptosis can play a leading role in certain anti-tumor treatments. Conversely, in normal tissues, pyroptosis contributes to treatment-related adverse effects. This further underscores the dual nature of pyroptosis.

### Pneumonia

As an open organ, the bronchial and alveolar epithelium of lung is exposed to air pollutants and pathogenic microbes. To defend against the invasion of pathogens, pattern recognition receptors (PRRs), which can be activated by PAMPs and DAMPs, are equipped with bronchial epithelial cells, dendritic cells, and alveolar macrophages [28, 61]. These receptors assemble inflammasomes with apoptosis-associated speck-like protein and procaspase-1. Inflammasomes occupy a key position in maintaining a delicate balance between immune responses and tissue injuries and/or infections. Once PRRs are activated, inflammasomes can induce pyroptosis and produce cytokines [28, 61] (Fig. 2).

Excessive inflammatory responses are always deemed to be an important cause of death following severe pneumonia. As a form of dysregulated hyperinflammation, cytokine release syndrome is the most significant cause of mortality in patients with severe pneumonia including COVID-19. Lactate dehydrogenase (LDH) and cytokines are highly elevated in these patients [62]. Growing evidence favors pyroptosis involvement [63]. Pyroptosis confers host protection to lung epithelial cells in some patients with infection [64–66]. Besides, pyroptosis, as an immune response against infections, plays a protective role in host defense in the initial period of pneumonia [67]. Nevertheless, excessive pyroptosis leads to tissue injury and host lethality [68]. In a mouse IAV infection model, persistent NLRP3 inflammasome activation leads to lung injury, which is not associated with viral titer [69]. Angiotensin-converting enzyme 2 (ACE2) is an important receptor of SARS-CoV-2 and a negative regulator of the renin-angiotensin-aldosterone system (RAAS) which is a hormone system that regulates blood pressure and fluid balance in the body. SARS-CoV-2 spike protein and cells expressing ACE2 form syncytia. Syncytia can activate the cascade from caspase-9 to caspase-3/7, resulting in GSDME-mediated pyroptosis [70]. Others have reported that the fusion activates NLRP3 inflammasome, which triggers GSDMD-mediated pyroptosis [71]. Non-Structural Protein 6 (NSP6) of SARS-CoV-2, as a key determinant of pathogenicity, is also reported to trigger GSDMD-mediated pyroptosis by activating NLRP3 inflammasome targeting ATP6AP1 [72, 73]. Some supportive evidences have even shown that angiotensin II elevated by RAAS can activate NLRP3 inflammasome [74–76]. The complement cascade can interact with SARS-CoV-2 and then be cleaved into fragments such as C3a and C5a anaphylatoxins. These fragments could also activate NLRP3 inflammasome. Coagulopathy is another life-threatening complication of SARS-CoV-2 and some influenza virus infections [77]. Proinflammatory cytokines, which can promote various procoagulation factors, are insufficient to explain the dramatic coagulation reaction. GSDMD-mediated pyroptosis can trigger blood clotting and cause massive thrombosis in both the canonical pathway and the non-canonical pathway [78]. This systemic fibrin deposition in the model of endotoxemia is reminiscent of coagulopathy seen in COVID-19.

In pneumonia caused by other pathogens, pyroptosis still plays an important role. Streptococcus pneumoniae can cause cell death by activating both an apoptotic pathway and a pyroptotic pathway. In the pyroptotic pathway, pneumoniae activates NLRP3 inflammasome to mediate IL-1β production and release through hydrogen peroxide produced by pneumococci as a product of the pyruvate oxidase SpxB [79]. In another study of H7N9 influenza, GSDME-mediated pyroptosis was revealed to contribute to the pulmonary cytokine storm of virus infection [80].

### Asthma

Asthma is a common chronic lung disease, that affects more than 300 million people worldwide [81]. Many studies indicate some correlation between asthma and pyroptosis. GSDMA and GSDMB on chromosome 17q are linked to asthma [82, 83]. The chromosome 17q region is the most consistently associated and powerful region with asthma susceptibility. In recent years, many studies have identified that GSDMB seems to play an important role in asthma susceptibility and severity. Single nucleotide polymorphisms in GSDMB display a strong correlation with GSDMB expression levels and the severity of asthma. Higher expression of GSDMB is correlated with antiviral pathways and exacerbations of asthma [84]. GSDMB has been demonstrated to be highly expressed in human asthmatic lungs, specifically in bronchial epithelial cells, but not to be significantly expressed in alveolar epithelial cells, fibroblasts, and smooth muscle [85, 86]. The overexpression of GSDMB can upregulate genes correlated with airway remodeling and airway hyperresponsiveness including 5-LO, TGF-β1, and MMP-9 [87]. A splice variant causing the deletion of exon 6, which encodes 13 amino acids in the N-terminal domain, has been reported to decrease the risk of asthma, suggesting that abolishing GSDMB-mediated pyroptotic activity may play a role in asthma [88]. However, at the present stage, direct proof of how pyroptosis affects asthma is still lacking.

### Other pulmonary diseases

Pyroptosis is reported to promote lung injury in acute respiratory distress syndrome (ARDS) [89]. Pyroptosis is also involved in other acute lung injuries (ALIs) such as brain injury-induced acute lung injury and pancreatitis-induced acute lung injury [90, 91]. Some natural products from various medicinal plants such as Emodin, Syringaresinol (SYR) and Honokiol (HKL), and 5-Androstenediol (5-AED), a natural steroid hormone can suppress pyroptosis to resist lung injury [92–95]. The pyroptosis of pulmonary artery smooth muscle cells can promote pulmonary hypertension [96]. Chronic obstructive pulmonary disease (COPD) is also considered to have a certain correlation with pyroptosis [97]. Additionally, nicotine has been found to influence the progression of COPD by GSDMD-induced pyroptosis [98].

### Therapeutic implications

Cell death facilitates maintaining physiological homeostasis and healthy development by removing cells suffering from damage or infection. Excessive cell death can also contribute to human pathologies [99]. The dual nature of pyroptosis has been a long-standing and intriguing topic. Pyroptosis is involved in the protection of the host, especially in the initial stage of some inflammatory respiratory diseases through immune defense. An excessive inflammatory response could cause ultimately tissue damage [61]. Uncontrolled pyroptosis is responsible for system-wide inflammation in a large number of lung inflammatory diseases. For lung cancer, from the perspective of inflammation, pyroptosis carries a potential risk of carcinogenesis. On the other hand, from the cell death perspective, pyroptosis can promote tumor cell death. At the same time, pyroptosis is also a significant factor causing adverse reactions in tumor therapy. Thus, how to harness this double-edged sword to yield more positive effects is worth our contemplation.

Currently, many basic trials have demonstrated that the specific compounds can directly or indirectly target pyroptosis. These compounds mainly include some common chemotherapy drugs, such as cisplatin, doxorubicin, and 5-FU [100]. These chemotherapy drugs have been applied in clinical treatment for decades. The pyroptosis recently discovered that the occurring alongside apoptosis in these drugs lacks sufficient specificity, but it is still meaningful. Knowledge of the pyroptosis pathway activated by these drugs can help optimize treatment strategies, overcome resistance, and reduce the side effects of chemotherapy. Other pyroptosis inducers are largely in the experimental stage including some natural products. From the perspective of drug development targeting pyroptosis, we are currently at the first step from bench to bedside.Precise drug delivery is another viable solution. A delivery platform based on macrophage was developed to achieve targeted tumor drug delivery and controlled release [101]. Some burgeoning nanoplatforms can also stably deliver drugs to activate pyroptosis [102]. Precisely activating pyroptosis in the tumor site can effectively prevent damage to normal tissues caused by pyroptosis.

A deep understanding of the pyroptosis pathway is also crucial. In specific diseases, only by understanding which mode of pyroptosis can exert a greater effect can better utilize pyroptosis for therapeutic benefits. For inflammatory diseases, the timing of pyroptosis inhibition is particularly crucial. Inhibiting pyroptosis too early might impact the early activation of the immune response. Conversely, inhibiting pyroptosis too late might not effectively curb the cytokine storm. For the clinical translation and application of pyroptosis, the deepening of theoretical knowledge about the pyroptosis pathway and the development of targeted drugs are both particularly important.

## Conclusion

**Fig. 3:**
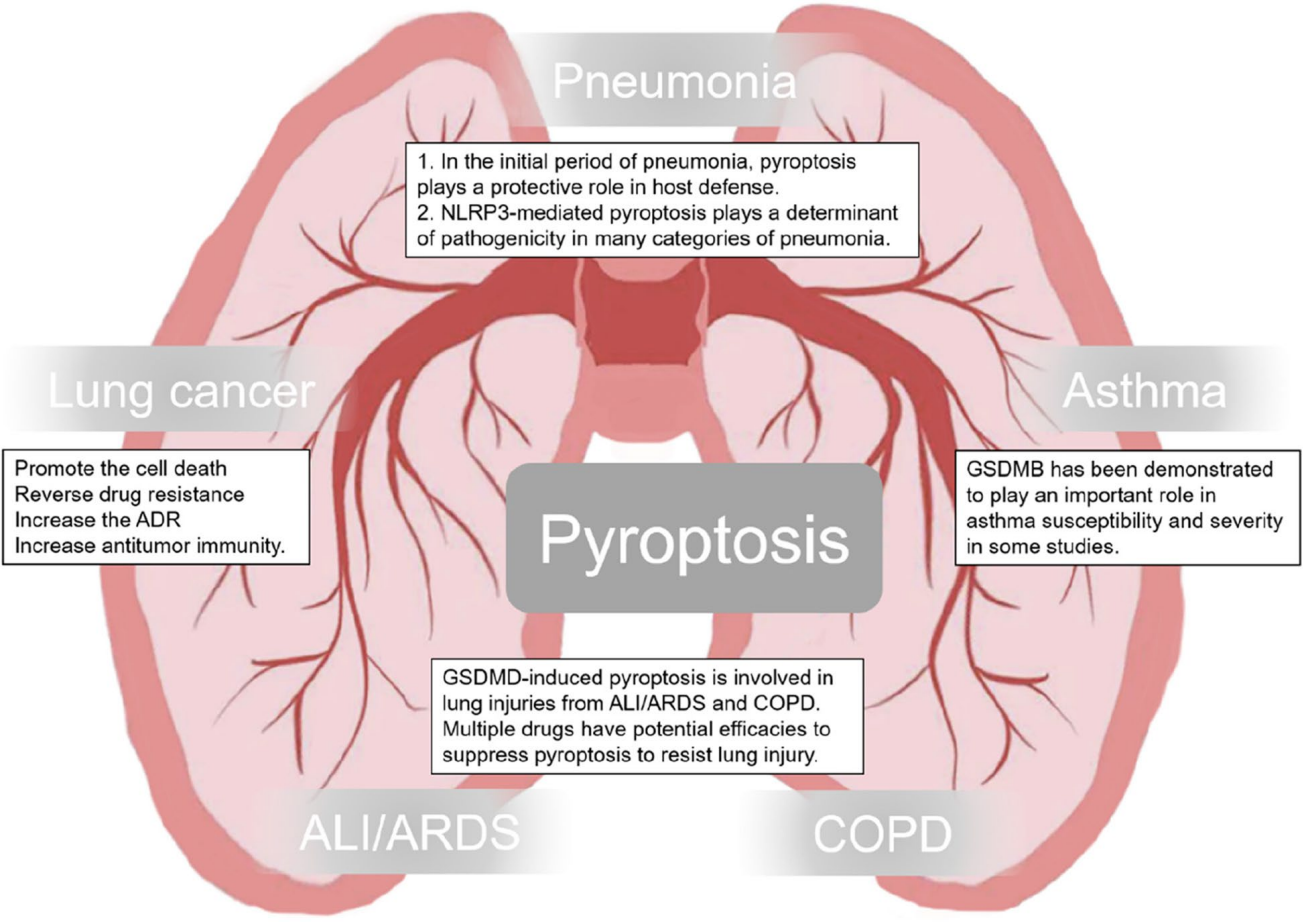
Role of pyroptosis in the lung. This schematic representation shows the important role of pyroptosis in the regulation of pulmonary diseases including pneumonia, asthma, lung cancer COPD, and ALI/ARDS

Pyroptosis, as a potential target for the treatment of pulmonary diseases, has gradually become clear. However, what needs to be emphasized is the dual nature of pyroptosis. For lung cancer, on the one hand, pyroptosis can promote the death of cancer cells and enhance antitumor immunity. On the other hand, pyroptosis also increases adverse drug reactions. For other pulmonary inflammatory diseases, pyroptosis can play both protective roles and negative roles in different periods of various diseases. Besides, pyroptosis has a potential effect on asthma (Fig. 3). In conclusion, pyroptosis is involved in a number of pulmonary diseases in different pathways. However, the understanding of pyroptosis in respiratory diseases is still limited. The future challenge lies in defining the details of the molecular mechanism of pyroptosis and how the details can improve the outcomes of diseases. The exploration of a balance point of pyroptosis for treatment should be a long-standing and open research area.

## Data Availability

No datasets were generated or analysed during the current study.
